# DIA-MS2pep: a library-free framework for comprehensive peptide identification from data-independent acquisition data

**DOI:** 10.52601/bpr.2022.220011

**Published:** 2022-12-31

**Authors:** Junjie Hou, Jifeng Wang, Fuquan Yang, Tao Xu

**Affiliations:** 1 National Laboratory of Biomacramolecules, CAS Center for Excellence in Biomacromolecules, Institute of Biophysics, Chinese Academy of Sciences, Beijing 100101, China; 2 Laboratory of Proteomics, Institute of Biophysics, Chinese Academy of Sciences, Beijing 100101, China; 3 Laboratory of Protein and Peptide Pharmaceuticals & Laboratory of Proteomics, Institute of Biophysics, Chinese Academy of Sciences, Beijing 100101, China; 4 College of Life Sciences, University of Chinese Academy of Sciences, Beijing 100049, China

**Keywords:** DIA-MS, Spectral library-free, Spectrum demultiplexing, Large precursor mass tolerance, Mass spectrometry

## Abstract

Identifying peptides directly from data-independent acquisition (DIA) data remains challenging due to the highly multiplexed MS/MS spectra. Spectral library-based peptide detection is sensitive, but it is limited to the depth of the library and mutes the discovery potential of DIA data. We present here, DIA-MS2pep, a library-free framework for comprehensive peptide identification from DIA data. DIA-MS2pep uses a data-driven algorithm for MS/MS spectrum demultiplexing using the fragments data without the need of a precursor. With a large precursor mass tolerance database search, DIA-MS2pep can identify the peptides and their modified forms. We demonstrate the performance of DIA-MS2pep by comparing it to conventional library-free tools in accuracy and sensitivity of peptide identifications using publicly available DIA datasets of varying samples, including HeLa cell lysates, phosphopeptides, plasma, *etc*. Compared with data-dependent acquisition-based spectral libraries, spectral libraries built directly from DIA data with DIA-MS2pep improve the accuracy and reproducibility of the quantitative proteome.

## INTRODUCTION

Data-independent acquisition (DIA), an alternative to data-dependent acquisition (DDA), has been an increasingly attractive method applied to mass spectrometry (MS)-based label-free proteomics due to its advantages in terms of quantification reproducibility, accuracy, and sensitivity (Chapman* et al.* 2014; Gillet* et al.* 2012; Hu* et al.* 2016; Zacchi and Schulz 2019; Zhang* et al.* 2020). In a DIA experiment, rather than with the precursor intensity-triggered mode in DDA, a mass spectrometer performs tandem MS/MS scans by fragmenting all the precursor ions within a series of predefined mass-to-charge (*m*/*z*) windows, so DIA MS theoretically records all the information of analytes in an unbiased way and is particularly beneficial for the detection and quantification of low-abundance peptides. However, identifying peptides and proteins from DIA data is not as straightforward as identifying them from DDA data due to the highly multiplexed MS/MS spectra and uncertain precursor-fragment relationships, which are not compatible with conventional DDA data search engines.

Developing more elaborate strategies for interpreting DIA data is imperative. Library-based search strategy has been widely adopted to detect peptides from DIA data (Rost* et al.* 2014). The spectral library can be either a sample-specific library from DDA experiments of the pooled sample, a species-specific library from the public peptide atlas resource (Rosenberger* et al.* 2014), or an *in silico* library constructed by predicting spectra from peptide sequences (Gessulat* et al.* 2019). However, these libraries can be expensive in terms of the time and amount of sample required, not reusable across laboratories or instrument platforms, or still immature for *in silico* spectra of the peptide with post-translational modifications (PTMs). Moreover, library-based peptide queries are limited to the depth of the library and cannot identify peptides with unexpected modifications or sequence variants.

Complementarily, several library-free tools, such as DIA-Umpire (Tsou* et al.* 2015), PECAN (Ting* et al.* 2017), directDIA (Bekker-Jensen* et al.* 2020a), PASS-DIA (Mun* et al.* 2020) and MaxDIA (Sinitcyn* et al.* 2021) have been developed to identify peptides from DIA data with no need of spectra library by directly searching the data against protein database. DIA-Umpire, a spectrum-centric tool, detects covarying precursor-fragment groups from DIA data to generate pseudo MS/MS spectra, which are then submitted to a conventional DDA search tool for peptide identification. DIA data are typically acquired by a single injection experiment, so the precursors, which are poorly detected or interfered with coeluting signals, are commonly observed, especially when analysing complex samples with limited chromatographic separation or samples with highly dynamic ranges of detectable concentrations. These will leverage the quality of pseudo-spectra generated by DIA-Umpire, as the spectra may contain insufficient peptide-specific fragments or ambiguous precursor masses and charges, leading to a low identification rate. PECAN, a peptide-centric method, performs the peptide query from DIA data against a background proteome database and reports the best evidence of detection and associated retention time. However, false positive detection evidence reported by PECAN may be introduced when the putative elution peak(s) of one peptide is shared by other peptides that have fragment(s) with an overlapping dot product distribution. In addition, PECAN does not provide scores for site-specific modifications and variant peptides. With a gas-phase fractionation (GPF) strategy (Ting* et al.* 2017), the peptide detection capability of library-free tools can be improved because the precursors in the MS1 scan are fragmented within narrower precursor isolation windows than those in a single-injection DIA experiment. However, GPF experiments require multiple injections of pooled samples, which may be impractical for cases with limited sample quantities, such as rare clinical samples.

To meet the above challenges, we present here a spectrum-centric framework, DIA-MS2pep, to identify peptides and their modified forms from DIA data in a library-free fashion. DIA-MS2pep uses a data-driven strategy for spectrum demultiplexing based on fragments data itself, which allows to effectively deconvolve the multiplexed spectra even when the signal of precursors is interfered or poorly detected. With a large precursor mass tolerance database search, DIA-MS2pep significantly improves the identification rate of pseudo-spectra generated from DIA data, and enables to identify the peptides containing post-translational modifications (PTMs). Using varying types of DIA datasets, we compare the performance of DIA-MS2pep with DIA-Umpire and PECAN in terms of accuracy and sensitivity of peptide identifications. Using the dataset of mixed proteome with a well-defined quantitative composition, we illustrate that, with DIA-MS2pep, the spectral library generated directly from DIA data allows to quantify the peptides and proteins with better precision than the DDA-based sample-spectral library. Lastly, when revisiting a real biological DIA dataset from HeLa cell proteome in response to serum starvation (Searle* et al.* 2018), using DIA data-specific DIA libraries built with DIA-MS2pep, we can quantify 25%–47% more differentially expressed proteins (*q*-value < 0.01) than that quantified using sample-specific DDA spectral library, and report hundreds of peptides with either chemical, biological modifications, or amino acid variants, offering a potentially valuable data resource for the further follow-up study.

## EXPERIMENTAL SECTION

### Workflow of MS2pep

#### MS2 spectrum self-demultiplexing

The Thermo MS. RAW files are converted into .mzML format using MSConvert (part of ProteoWizard (Chambers* et al.* 2012), v3.0.9974) with MS1 and MS2 vendor peak picking enabled, 64-bit binary precision and other default options. The .ms1 and .mgf files are subsequently converted from .mzML files using MSConvert or an in-house Perl script.

DIA-MS2pep extracts the chromatographic features of fragments using a fixed number of MS2 spectra data, which is calculated as the average time that one certain precursor is consecutively observed as a base peak in MS1 scan. After obtaining the information of the isolation window setting from the .mzML file, DIA-MS2pep then reads the MS2 spectra from .mgf files to carry out data processing for self-demultiplexing:

1　A data matrix of the fragment ion intensity (*I*) and scan cycle (*C*) is constructed from the fixed number of MS2 spectra (S) acquired by the same isolation window. Here, we take five fixed numbers (S = 5) as an example,

$ \;\;\;\;\;\;\begin{aligned} {\;}\\ F_{1, c 0}\\ F_{2, c 0}\\ F_{3, c 0} \\ \vdots \\ F_{n-2, c 0} \\ F_{n-1, c 0}\\ F_{n, c 0}\end{aligned}
\left. \begin{aligned}
 &  \quad C_{-2} \quad\quad\;\;\; C_{-1} \quad\quad\;\;\; C_{0} \quad\quad\;\;\; C_{1} \quad\quad\;\; C_{2} \\
  & \begin{array}{|ccccc|} \hline
I_{f 1, c-2} & I_{f 1, c-1} & I_{f 1, c 0} & I_{f 1, c 1} & I_{f 1, c 2} \\
I_{f 2, c-2} & I_{f 2, c-1} & I_{f 2, c 0} & I_{f 2, c 1} & I_{f 2, c 2} \\
 I_{f 3, c-2} & I_{f 3, c-1} & I_{f 3, c 0} & I_{f 3, c 1} & I_{f 3, c 2} \\
 \vdots & \vdots & \vdots & \vdots & \vdots \\
 I_{fn-2, c-2}  & I_{f n-2, c-1} & I_{f n-2, c 0} & I_{f n-2, c 1} & I_{f-2, c 2} \\
I_{f n-1, c-2} & I_{f n-1, c-1} & I_{f n-1, c 0} & I_{f n-1, c 1} & I_{f n-1, c 2} \\
 I_{f n, c-2} & I_{f n, c-1} & I_{f n, c 0} & I_{f n, c 1} & I_{f n, c 2} \\\hline
\end{array}
\end{aligned}\right.$

　 where *C*_0_ is the spectrum to be demultiplexed, and C_–__1_, C_–2_, C_1 _and C_2 _represent the spectra of the preceding and following two cycles, respectively. *F*_*n,c*0_ represents the *N*th fragment in the spectrum (*C*_0_). In the spectrum (*C*_0_), the fragments with *m*/*z* values less than 140 or within the *m*/*z* range of the isolation window are excluded as the default. Given that there are 100 fragments in C_0_, DIA-MS2pep individually extracts the fragment signal of the same *m*/*z* within the mass tolerance from the spectrum of C_–1_, C_–2_, C_1_ and C_2_, and finally builds a data matrix containing 5 × 100 intensities as above, where *I*_*f,c*_ is the intensity of a given fragment (*f*) in the spectrum of the scan cycle (*c*).

2　The detection of the base peak in the spectrum (*C*_0_). Apart from the highest intensity, one fragment considered by DIA-MS2pep as the base peak should also meet three additional criteria: (1) the peak has an *m/z* value larger than 300; (2) it has a non-zero intensity in at least three consecutive spectra including *C*_0_; and (3) it has an intensity higher than 1% of the intensity of the base peak in the raw MS2 data.

3　The Pearson correlation coefficient (PCC) of the intensity profiles between the base peak and the other fragments (base peak-fragment correlations) is calculated. If the correlation vector of fragments with a PCC above 0.9 is more than ten, DIA-MS2pep will continue the following steps of spectrum demultiplexing. Otherwise, DIA-MS2pep will stop the step of demultiplexing.

4　The resulting data points of PCC are submitted to one-dimensional kernel density estimation (KDE), which is performed using the Perl module “Statistics::KernelEstimation”. A Gaussian kernel is used for the distribution estimation of the PCC data, and the kernel bandwidth is set to 0.05 by default. DIA-MS2pep enables to find the boundary between the Gaussian distributions and determine the corresponding PPC (*p*), and determine the threshold of the PCC value (*p*_0_) as MAX{*p,* 0.8}, where 0.8 is the minimal PCC value of base peak-fragment correlations required by DIA-MS2pep (supplementary Fig. S1a and S1b).

5　Generation of the pseudo-spectrum in a two-step way (supplementary Fig. S1c). First, the fragments with a PCC value above *p*_0_ are considered as the 1st collection of fragments (*F-*1*st*) in the pseudo-spectrum. Second, DIA-MS2pep performs another round of PCC calculations between the fragments in *F-*1*st* and the others. Fragments with a PCC above *p*_0 _are considered as the 2nd collection of fragments (*F-*2*nd*). Finally, *F-*1*st* and *F-*2*nd* are together reported as a pseudo-spectrum.

6　The remaining fragments (*F-rest*) are treated as the input for Step Two in the next iteration unless the number of *F-rest* is smaller than ten.

7　If no pseudo-spectrum is generated by the above steps (1–6), then we still report the raw data as a pseudo-spectrum.

All the pseudo-spectra are stored in .mgf format, in which the center *m/z* value of the precursor isolation window is assigned as the pseudo-precursor *m/z*.

#### Large precursor mass tolerance database searching

DIA-MS2pep performs a large precursor mass tolerance database search using MSFragger (v.2.4), a DDA search engine used for open search. The pseudo-spectra are repeatedly searched by assigning different charge states (1–5 as default) to the precursor. DIA-MS2pep sets the precursor mass tolerance for the MSFragger database search in two ways:

1 Charge-dependent

precursor_mass_lower = $ -\left(\dfrac{w*z}{2}+3\right) $,

precursor_mass_upper = $ \dfrac{w*z}{2}+3 $,

where *w* is the isolation window size (Da), *z* is the pseudo-charge state, and the addition of 3 Da aims to cover putative precursor isotopes that might be splitting between isolation windows.

2 Charge-independent

precursor_mass_lower = −100,

precursor_mass_upper = 400.

These two modes are user-defined in a mutually exclusive way. Normally, compared with the charge-independent mode, the charge-dependent mode reports slightly more peptide identifications with less running time due to smaller database searching space. It is recommended to choose a charge-dependent mode for the DIA data from PTM-enriched samples.

#### Search data refinement

DIA-MS2pep performs the following steps to sequentially check the confidence of peptide identification and remove false-positive peptide hits.

1　It is determined whether a real precursor signal matches the theoretical mass of the peptide hit (within the mass tolerance of the instrument) in the MS1 scan. If so, then the number of isotopic peaks is traced as an auxiliary peptide feature. If multiple peptide hits share the same precursor, then DIA-MS2pep keeps the one with a search score (Expect score reported by MSFragger) of higher confidence.

2　If no precursor evidence is found in the above step, then DIA-MS2pep determines whether the peptide can be interpreted as having a potential modification by performing a putative modification search (for details, see the “Putative modification analysis” section below).

3　DIA-MS2pep estimates the mass accuracy by calculating the mean value (*m*) and standard deviation (*s*) of the precursor in ppm using the verified peptide hits with an expect score of less than 0.01 and then using the mass deviation (*m* ± 3*s*) as a filter to remove the false-positive peptide hits.

4　Not all the mass shifts reported using open search by MSFragger represent true modifications due to possible artifacts from unaccounted missed cleavages or co-fragmentation (Chang* et al.* 2020). For the situation in which one spectrum reports multiple peptides, using the basic idea of Crystal-C (Chang* et al.* 2020), DIA-MS2pep will check whether the difference among these peptide hits is caused due to the missed enzymatic cleavage. If so, DIA-MS2pep only keeps the one scored with the highest confidence.

5　If two or more peptide hits are identified by the same pseudo-spectrum, with homologous sequences with no more than two different amino acids, then DIA-MS2pep only retains the one with a search score of higher confidence.

#### FDR estimation

DIA-MS2pep employs a target-decoy approach to control the false discovery rate using Percolator. DIA-MS2pep calculates the auxiliary peptide features to improve the performance of the Percolator for the discrimination of target and decoy peptide hits (supplementary Fig. S2). For PTM-enriched data, such as the phosphoproteome, DIA-MS2pep performs FDR estimation of peptides with or without modifications separately. The peptide scores of modified peptides, such as phosphopeptides, are normally lower than those of unmodified counterparts (Du* et al.* 2008).

### Putative modification analysis

DIA-MS2pep performs a putative modification search against the Unimod database (Creasy and Cottrell 2004). By default, DIA-MS2pep considers all the modifications listed in the Unimod database, and also allows users to define their own modifications of interest into Unimod.xml files. The basic idea of this search is to determine the probability of interpreting the MS1 signals detected within the isolation window as a precursor of a peptide with a putative modification, in which the mass matches the difference between the theoretical mass of the peptide and the mass of the signal in MS1 within the precursor mass tolerance.

#### Precursor signal filtering

Because the signals in the MS1 scan are highly complex, before considering them as candidates for putative modification analysis, they should pass the following stringent criteria: (1) a signal-to-noise ratio above 10; (2) observation of at least two consecutive MS1 spectra; (3) a number of isotopic peaks greater than 3; and (4) a Pearson correlation coefficient of the intensity patterns between the theoretical and observed isotopic peaks larger than 0.8. Here, theoretical isotope peak patterns (Kubinyi 1991) are calculated using stripped peptide sequences.

#### Modification score calculation

DIA-MS2pep searches the potential modification candidates against the Unimod database and calculates the modification score and site localization probability by implementing a similar algorithm as ptmRS (Taus* et al.* 2011). A site probability score > 0.75 is required for data processing in the next step.

#### Precursor-fragment correlation

To remove as many false-positive matches as possible, DIA-MS2pep further calculates the PCC of the intensity profile between the candidate precursor and at least four matched fragments (from the top five candidates ranked by intensity). A candidate is required to have a median PCC greater than 0.9.

#### Rank of candidates

DIA-MS2pep ranks candidates with putative modifications based on: (1) the length of the sequences consecutively matched b- and y- ions; (2) the intensity of the precursor isotopes; and (3) the median of the PCC in the last step. The top-ranked candidate is finally reported as a peptide with a corresponding putative modification.

### Phosphorylation site analysis

For comparison with the phosphorylation site confidence calculated by DIA-MSpep, DDA data (and pseudo-spectra generated by DIA-Umpire) were searched using Sequest HT (Eng* et al.* 1994) in Proteome Discoverer (PD) 1.4 software, which used Percolator to report peptide identifications with a *q*-value < 0.01. The site localization confidence was evaluated by PhosphoRS (Taus* et al.* 2011) Node embrace in PD 1.4. Sites with a localization probability > 0.75 were considered confident results.

### DIA dataset and protein sequence databases

All the DIA datasets used in this study are previously published data, of which the detailed information is listed in supplementary Table S1. The protein sequences used for the database search were downloaded from the UniProt proteome (access date: 2020.03.10): *H. sapiens* (44,254 entries), *C. elegans* (26,927), *S. cerevisiae* (6,049 entries) and *E. coli* (4,391). Decoy protein sequences are generated by randomizing target protein sequences using the shuffle strategy.

### Parameters setting of library-free tools

For DIA-MS2pep, the entire pipeline of DIA-MS2pep contains four components: DIA/SWATH_pesudo_MS2, MSFragger_runner, DIA/SWATH_data_refinement and percolator_runner. The pseudo-spectra are first generated by DIA/SWATH_pesudo_MS2, and then searched by MSFragger_runner, which implements MSFragger v2.4 in the current study. The precursor mass range is automatically determined based on the isolation window setting in DIA experiment. The resulting PSMs are refined by DIA/SWATH_data_refinement as described in the supplementary Methods. The refined peptide hits from both target and decoy proteins are stored in a PIN-format file as input of Percolator (v3.02.1) for the validation at the 1% FDR of PSM, peptide and protein levels. For phosphopeptides, DIA-MS2pep automatically calculates the site confidence score as PhosphoRS (Taus* et al.* 2011), and localized phosphopeptides are filtered with a localization probability > 0.75.

For DIA-Umpire, if available, we directly used the pseudo-spectra from the original manuscript, including the HeLa_DIA and PhosphoHeLa_DIA datasets. Otherwise, for the Plasma_GPF_DIA and PhosphopPep_DIA datasets, we generated pseudo-spectra via DIA-Umpire (v2.1.3) with its sample parameter file by the default option, which is deposited into ProteomeXchange (Vizcaino* et al.* 2014).

For PECAN, we use its alternative method Walnut, which is an implementation of the PECAN scoring system in EncyclopeDIA (Searle* et al.* 2018) (version 0.9.5). The search parameters were set as described in PECAN’s manuscript: precursor and fragment tolerance: 10 ppm; fragmentation: HCD (Y-Only); Percolator version: v3-01; enzyme: trypsin; DIA acquisition type: non-overlapping DIA; Target/Decoy Approach: Normal; and charge range: 2 to 3.

### Protein quantification analysis

#### Library generation

We used the confident peptides reported by Percolator (*q*-value < 0.01) to create a BiolioSpec-supporting input file (Frewen* et al.* 2006). SSL (spectrum sequence list) containing file, scan, charge and sequence information. Then, with the .SSL files as the input, DIA data-specific spectral libraries for MultiOrg_DIA and HeLa_Serum_DIA datasets were created from DIA raw data using the BlibBuild tool embraced in Skyline software (MacLean* et al.* 2010) (v19.1.0.193). For the HeLa_Serum_DIA dataset, four DIA libraries were generated: DIA-MS2pep_Lib from 36 wide-window (24 Da) DIA data and DIA-MS2pep_GPF_Lib from 6x GPF narrow-window (4 Da) DIA data plus DIA-MS2pep_Lib.

#### Quantification with EncyclopeDIA

The resulting BLIB library was converted into a chromatogram library by EncyclopeDIA (Searle* et al.* 2018) (version 0.9.5) and used to search the mzMLs to quantify peptides and proteins. The search parameters of EncyclopeDIA were configured as follows: precursor, fragment, and library tolerance: 5 ppm for MultiOrg_DIA and 10 ppm for HeLa_Serum_DIA; fragmentation: both b- and y-ions; the number of quantitative ions: 5; minimum number of quantitative ions: 3; Percolator version: v3-01; enzyme: Trypsin; DIA acquisition type: non-overlapping DIA for MultiOrg_DIA and overlapping DIA for Serum_HeLa_DIA; Target/Decoy Approach: Normal; and background: the mixed four species protein database for MultiOrg_DIA and the human protein database for HeLa_Serum_DIA. Protein quantities were calculated as the sum of peptide quantities. Specifically, for the HeLa_Serum_DIA dataset, we followed the same criteria for data filtering as described in the original manuscript (Searle* et al.* 2018): the peptides need to be measured in every replicate of at least one time-point and with cross experiment CVs less than 20%. For the quantitative data from the HeLa-specific DDA library (DDA_Lib), we used supplementary Data 1 from the original manuscript of EncyclopeDIA (Searle* et al.* 2018).

### Bioinformatics analysis

Hierarchical clustering analysis (by the “pheatmap” R package), protein quantification data normalization of HeLa_Serum_DIA using the method of Remove Unwanted Variation Using Residues (RUVr (Risso* et al.* 2014), by the “RUVSeq” R package) followed by differential gene expression analysis (by the “edgeR” R package (Lund* et al.* 2012)) and gene set enrichment analysis (GSEA) with the Reactome pathway database (by the “fgsea” R package (Sergushichev 2020)) were performed in R, Windows Rx64, version 3.5.2.

### Data and code

The pseudo-spectra (.mgfs) generated by DIA-MS2pep from the DIA datasets; the resulting files, .pepXML from MSFragger, .msf from Proteome Discover 1.3, and .pin from DIA-MS2pep, and the Percolator-reported peptides and proteins files (.pin.target.pep.tsv and .pin.protein.tsv); and the spectrum library (.dlib and .elib) of MultiOrg_DIA and HeLa_Serum_DIA dataset have been deposited to the ProteomeXchange Consortium (Vizcaino* et al.* 2014) (http://proteomecentral.proteomexchange.org) via the iProX partner repository (Ma* et al.* 2019) with the dataset identifier PXD032253. The DIA-MS2pep source code and its documentation are freely available at https://github.com/SS2proteome/DIA-MS2pep.

## RESULTS AND DISCUSSION

### Framework of DIA-MS2pep

**Figure 1:**
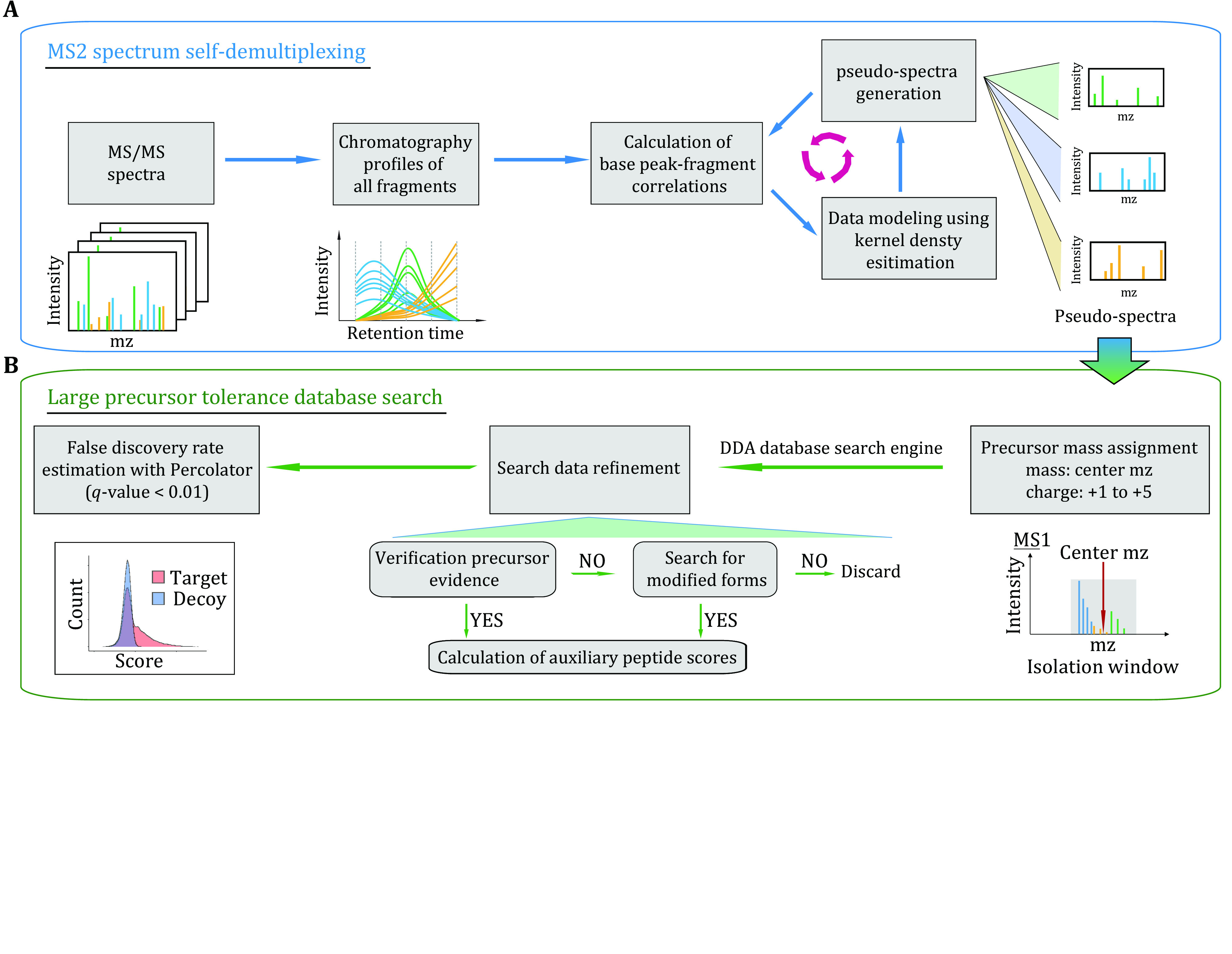
Framework of DIA-MS2pep. **A** DIA-MS2pep iteratively generates the pseudo-spectra from DIA data by spectrum self-demultiplexing using MS2 data only. **B** The pseudo-spectra are assigned with the center *m*/*z* of the isolation window and searched with the DDA search engine using a large precursor mass tolerance strategy. With rigorous data refinement, including verification precursor evidence, searching for modified forms and computation of auxiliary peptide scores, all the target and decoy peptide hits are submitted to the Percolator to estimate the false discovery rate and report peptide and protein results with a *q*-value < 0.01

DIA-MS2pep comprises two main components for peptide identification from DIA data: MS2 spectrum self-demultiplexing (Fig. 1A) and large precursor mass tolerance database search (Fig. 1B). In brief, after extracting the chromatographic profiles of fragments, DIA-MS2pep demultiplexes the MS2 spectrum in a recurrent way: the algorithm iteratively performs data modelling of base peak-fragment correlation (Pearson correlation coefficient of the intensity profile between the peak of the highest intensity and the remaining peaks in the MS2 data) with one-dimensional kernel density estimation (KDE), “pops-out” fragments with a chromatography profile close to that of the base peak as pseudo-spectra, and keeps the remaining fragments as the input for the next iteration. Each resulting pseudo-spectrum is assigned with the centre mass of the isolation window as pseudo-precursor mass, and then searched using a large precursor mass tolerance strategy by MSFragger (Kong* et al.* 2017). Subsequently, DIA-MS2pep performs the data refinement of search results to keep the peptide identifications that are either verified by the evidence of supporting precursors in MS1 scan or annotated as the modified peptides. DIA-MS2pep also includes the step for modification site localization scoring. Finally, Percolator (Spivak* et al.* 2009) is employed to rank the resulting collection of peptide spectrum matches (PSMs), using both peptide scores reported by the DDA search engine and additional auxiliary peptide features (supplementary Table S2) computed by DIA-MS2pep, and it reports the peptide identifications with a false discovery rate (FDR) of 1% at the unique peptide and protein level.

### The rationale of spectrum self-demultiplexing

One of the key steps for a spectrum-centric method is to extract sufficient peptide-specific fragment ions from multiplexed spectra of DIA data. For current spectrum-centric tools, such as DIA-Umpire, the detection of covarying precursor-fragment ion groups is the main principle for demultiplexing DIA MS2 spectra. However, poor precursor-fragment correlations caused by the lack of a detectable precursor signal or signal interference from co-eluted peptides are common phenomena in DIA MS, and this is further amplified for short chromatographic gradients or samples containing highly abundant protein/peptides, such as plasma or serum. DIA-MS2pep addresses this bottleneck using a data-driven method based on MS2 data itself without the need of precursor information.

We first validate the rationale of MS2 spectrum self-demultiplexing using four previously published DIA datasets: HeLa_DIA, Plasma_GPF_DIA, HeLa_gradient_DIA and MultiOrg_DIA (supplementary Table S1). These datasets were collected by different DIA experiment settings or sample properties, where HeLa_DIA for different isolation windows, Plasma_GPF_DIA for high dynamic content in the sample, HeLa_gradient_DIA for different lengths of LC gradient and MultiOrg_DIA for complex peptides mixture from different species. We first compute the data points per peak of both precursors and fragments from the peptides identified in each DIA dataset (supplementary Fig. S3a and S3b**)**. The results show that fragment ions can be detected with more data points across chromatographic peaks than precursor ions and are not affected by the sampling rate of the DIA methods (HeLa_DIA), the dynamic range of sample quantity (Plasma_DIA), the length of LC gradient (HeLa_gradient_DIA) or complex proteome sample (MultiOrg_DIA). These findings indicate that even though no precursor is detected in the MS1 scan, fragment ions are still detectable in the MS2 scan. We next compare the distribution of the precursor-fragment correlations and intra-fragment correlations (supplementary Fig. S3c and S3d). Here, for intra-fragment correlation, we calculate the Pearson correlation coefficient (PCC) of the LC elution profiles between the base peak and the rest of the peaks matched to the peptide in an MS2 spectrum, namely, the base peak-fragment correlation. For the HeLa_DIA dataset, the base peak-fragment correlation is significantly higher than the precursor-fragment correlation, especially for data with fewer data points per peak (5 Da). For the Plasma_DIA dataset, even with the GPF strategy, by which the median data points per peak of the precursor is 13, the median precursor-fragment correlation is still less than 0.9, while the median base peak-fragment correlation is as high as 0.99. These results indicate that coeluted highly abundant peptides could interfere/suppress the MS signal of the target peptide more significantly at the MS1 level than at the MS2 level, thus leading to an inconsistent precursor MS signal. For HeLa_gradient_DIA and MultiOrg_DIA datasets, we also observe that base peak-fragment correlations consistently are higher than precursor-fragment correlations. Taken together, the base peak-fragment correlation has great potential for DIA MS2 spectrum demultiplexing.

### Performance evaluation of spectrum self-demultiplexing

**Figure 2:**
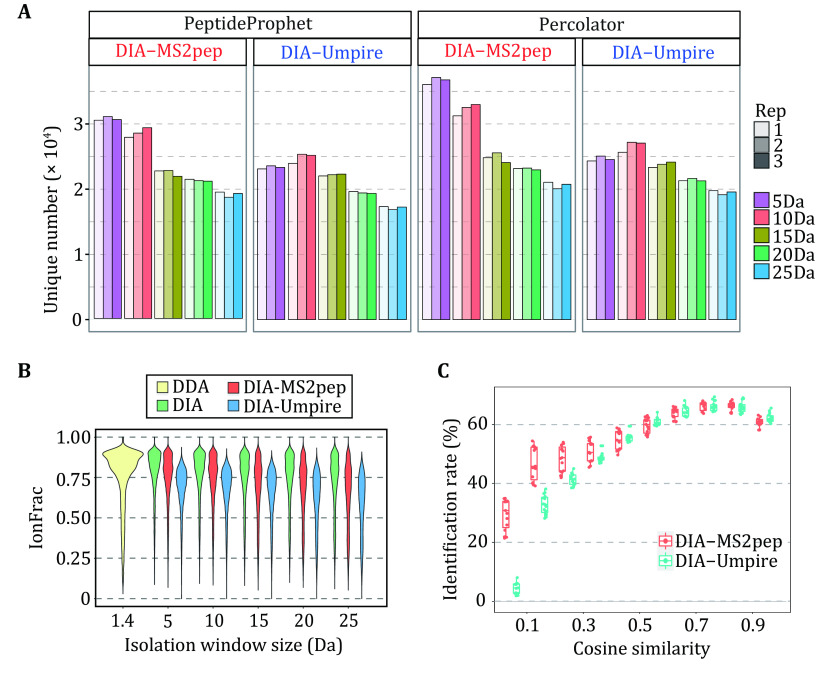
Performance evaluation of spectrum self-demultiplexing. **A** The comparison of the number of unique peptides identified from the HeLa_DIA dataset using DIA-MS2pep and DIA-Umpire with 1.0% of FDR estimated using either PeptideProphet or Percolator. **B** The fractions of matched fragments in DDA spectra, DIA spectra and pseudo-spectra generated by DIA-MS2pep and DIA-Umpire, are calculated as the longest peptide sequence covered by consecutive b- or y-ions divided by the peptide length. The peptide ions for violin plotting are identified from DDA data and pseudo-spectra generated DIA-MS2pep and DIA-Umpire in common (*n* = 8556). **C** The identification rate as a function of cosine similarity of pseudo-spectra generated by DIA-MS2pep and DIA-Umpire from one given DIA spectrum

Using a HeLa cell lysates DIA dataset containing 15 MS runs from five different DIA experiments with different isolation window sizes (HeLa_DIA dataset (Tsou* et al.* 2016)), we first test the capability of peptide identification of DIA-MS2pep relative to DIA-Umpire. For DIA-Umpire, we adopt the pseudo-spectra in the original manuscript (Tsou* et al.* 2016), and keep both the search setting and peptide FDR estimation method as close as possible to DIA-MS2pep. With a 1% FDR of unique peptides evaluated by either PeptideProphet or Percolator (supplementary Fig. S4 and Fig. S5). DIA-MS2pep can identify more peptides than DIA-Umpire from DIA data (Fig. 2A), particularly those collected using the narrow isolation window size (5 Da and 10 Da).

The performance of spectrum demultiplexing by DIA-MS2pep is next evaluated by calculating the fraction of peptide fragment ions matched in pseudo-spectra, raw DIA spectra and DDA spectra (Fig. 2B). Not surprisingly, compared with DIA spectra, DDA spectra contain a higher fraction of peptide-specific fragments due to MS2 data collected with the narrowest isolation window (1.4 Da). The pseudo-spectra generated by DIA-MS2pep contains the peptide-specific ions closer to the raw DIA spectra than those generated by DIA-Umpire. Furthermore, we investigate the identification rate as a function of cosine similarity between the pseudo-spectra that are generated by either DIA-MS2pep or DIA-Umpire from one given DIA spectrum. As illustrated in Fig. 2C, for two types of pseudo-spectra, their identification rates are very close when their cosine similarities are high; in contrast, if their cosine similarities are low, pseudo-spectra generated by DIA-MS2pep yield a significantly higher identification rate than those generated by DIA-Umpire. Therefore, these results demonstrated that DIA-MS2pep outperforms DIA-Umpire in the spectrum demultiplexing of DIA data.

### Peptide identification with large precursor mass tolerance database search

For the spectrum-centric approach, such as DIA-Umpire, either precursor ions in MS1 scan, that are highly correlated with fragments, or unfragmented precursor ions in MS2 scan are used for the database search using the DDA search tool with the strict precursor mass mode. However, DIA data lack of a direct relationship between the precursor and its fragment ions, and the accuracy of the peptide precursor including both *m*/*z* and charge, especially for low-abundance peptides, is still a critical aspect that requires thorough investigation. To address this issue, DIA-MS2pep performs the database search of pseudo-spectra using the center *m*/*z* in the isolation window as a pseudo-precursor with a large precursor mass mode, and then verifies the precursor evidence of each PSM during the post-refinement of search data. It is important to note that the step of verifying the precursor signal is necessary to reduce the false-positive peptide hits (supplementary Fig. S6).

To investigate the performance of peptide identification with a large precursor mass tolerance search by DIA-MS2pep, we design a simulation experiment as illustrated in supplementary Fig. S7a. In brief, the pseudo-spectra generated by DIA-Umpire from the HeLa_DIA dataset are searched by MSFragger with either strict precursor mass or large precursor mass. For the latter mode, the pseudo-spectra are first modified by replacing the precursor mass originally assigned by DIA-Umpire as the center *m*/*z* of the isolation window to mimic the pseudo-spectra generated by DIA-MS2pep. To facilitate a fair comparison, the data resulting from two search modes are submitted to DIA-MS2pep pipeline for the data refinement and FDR estimation with Percolator. Compared with the strict precursor mass search, a large precursor mass tolerance search dramatically improves the identification rate of the pseudo-spectra (supplementary Fig. S7b). In addition, looking into the details of the search results, we find that those pseudo-spectra are not identified as PSMs using strict precursor mass search but rescued by large precursor mass search, commonly contained sufficient fragment ions but they contain the incorrect precursor m/z or charge state assigned by DIA-Umpire (supplementary Fig. S7c). Therefore, a large precursor mass search is an effective strategy for the peptide identification from pseudo-spectra generated from DIA data.

### Performance evaluation of DIA-MS2pep on GPF DIA data

**Figure 3:**
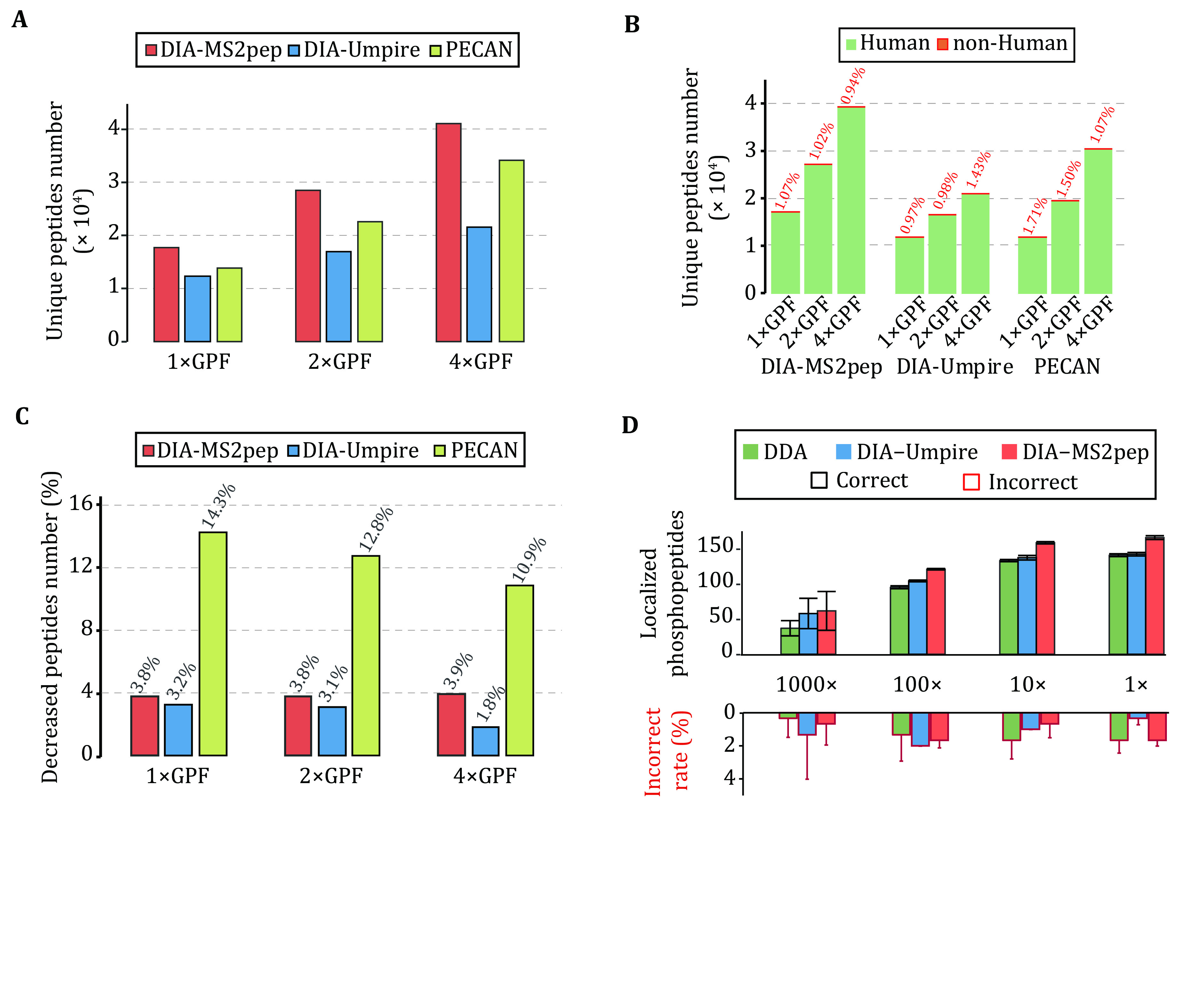
The valuation of DIA-MS2pep using HeLa_GPF_DIA and PhosphopPep_DIA dataset. **A** The unique peptide number identified from the HeLa_GPF_DIA dataset by DIA-MS2pep, DIA-Umpire and PECAN. **B** The unique peptide numbers reported by DIA-MS2pep, DIA-Umpire and PECAN against four species databases (*H. sapiens*, *C. elegans*, *S. cerevisiae* and *E. coli*). The percentage of peptides not from *H. sapiens* is labelled (red). **C** The percentages of the decrease in peptide numbers reported by DIA-MS2pep, DIA-Umpire and PECAN search against the four species databases relative to that against the *H. sapiens* database only. **D** The number of correctly localized phosphopeptides (200 synthetic peptides in total) identified from a diluted yeast background (PhosphopPep_DIA dataset) (Bekker-Jensen* et al.* 2020a)

It was demonstrated that improved precursor selectivity with the GPF strategy dramatically improves the performance of both DIA-Umpire and PECAN in peptide detection from DIA data (Ting* et al.* 2017). We also evaluate the performance of DIA-MS2pep using GPF DIA datasets (HeLa_GPF_DIA). In total, DIA-MS2pep identifies 17,853, 28,615 and 41,217 unique peptides from the 1×GPF, 2×GPF and 4×GPF datasets, which are 3,999, 5,852, and 6,916 more than PECAN, and 5576, 11565 and 19630 more than DIA-Umpire, respectively (Fig. 3A). Of the 16,596 common peptides identified in three GPF DIA data, 0.3% of peptides (close to 0.2% reported by PECAN) show a discrepancy of the retention time in either the 2×GPF or 4×GPF dataset compared with the 1×GPF dataset (supplementary Fig. S8).

To evaluate the accuracy of peptide identifications, using the same idea of the “Entrapment” strategy (Granholm* et al.* 2011), we challenge DIA-MS2pep, DIA-Umpire and PECAN by searching the HeLa_GPF_DIA dataset against a more complex protein database containing four species, *H. sapiens, E. coli, C. elegans* and* S. cerevisiae*. The results show that, on average, PECAN reports a higher percentage of non-human peptides than DIA-MS2pep and DIA-Umpire (Fig. 3B). Moreover, searching with a complex database leads to more decrease in peptide identification reported by PECAN than that reported by either DIA-MS2pep or DIA-Umpire (Fig. 3C). These results demonstrate that DIA-MS2pep outperforms PECAN in terms of sensitivity and accuracy of peptide identification from DIA data.

### Identifying the peptides with PTMs from DIA data

With the benefit of a large precursor mass tolerance search strategy, DIA-MS2pep is able to identify the peptides with un-predefined PTMs. For peptide hits for which the theoretical precursor was not found in MS1 scan, DIA-MS2pep attempts to determine the probability that one certain precursor signal can be annotated as the peptide with a putative modification, from a user-defined list or the Unimod database (Creasy and Cottrell 2004), and localize the modification site with a confidence score using the algorithm adapted from ptmRS (Taus* et al.* 2011), which is a site localization tool for DDA data (more details in Methods).

Using the DIA datasets of synthetic phosphopeptides (200 species) spiked with a stable background of tryptic yeast phosphoproteome samples at different concentrations (Bekker-Jensen* et al.* 2020a) (PhosphopPep_DIA dataset), we first test the accuracy of the pseudo-spectra generated by DIA-MS2pep for the localization of modification sites. Consistent with previous criteria (Taus* et al.* 2011), DIA-MS2pep reports the phosphopeptides with at least 0.75 site confidence as correct localization. Compared with DIA-Umpire, DIA-MS2pep identifies more correctly localized phosphorylation sites from synthetic phosphopeptides on average, which is also more than that identified from DDA data. DIA-MS2pep reports on average 0.97% of incorrectly assigned sites, lower than 1.29% for DIA-Umpire and 1.18% for DDA data (Fig. 3D and, supplementary Table S3). When analysing more complex phosphopeptides from serum-stimulated HeLa cells (Searle* et al.* 2019), DIA-MS2pep identifies 70% more phosphopeptides in total and localizes 78% more phosphopeptides than DIA-Umpire (supplementary Fig. S9). These results demonstrate that the pseudo-spectra extracted from DIA data by DIA-MS2pep contain sufficient fragment ions for the identification of peptides with PTMs, such as phosphorylation, and are compatible with the DDA localization tool for the evaluation of site localization.

**Figure 4:**
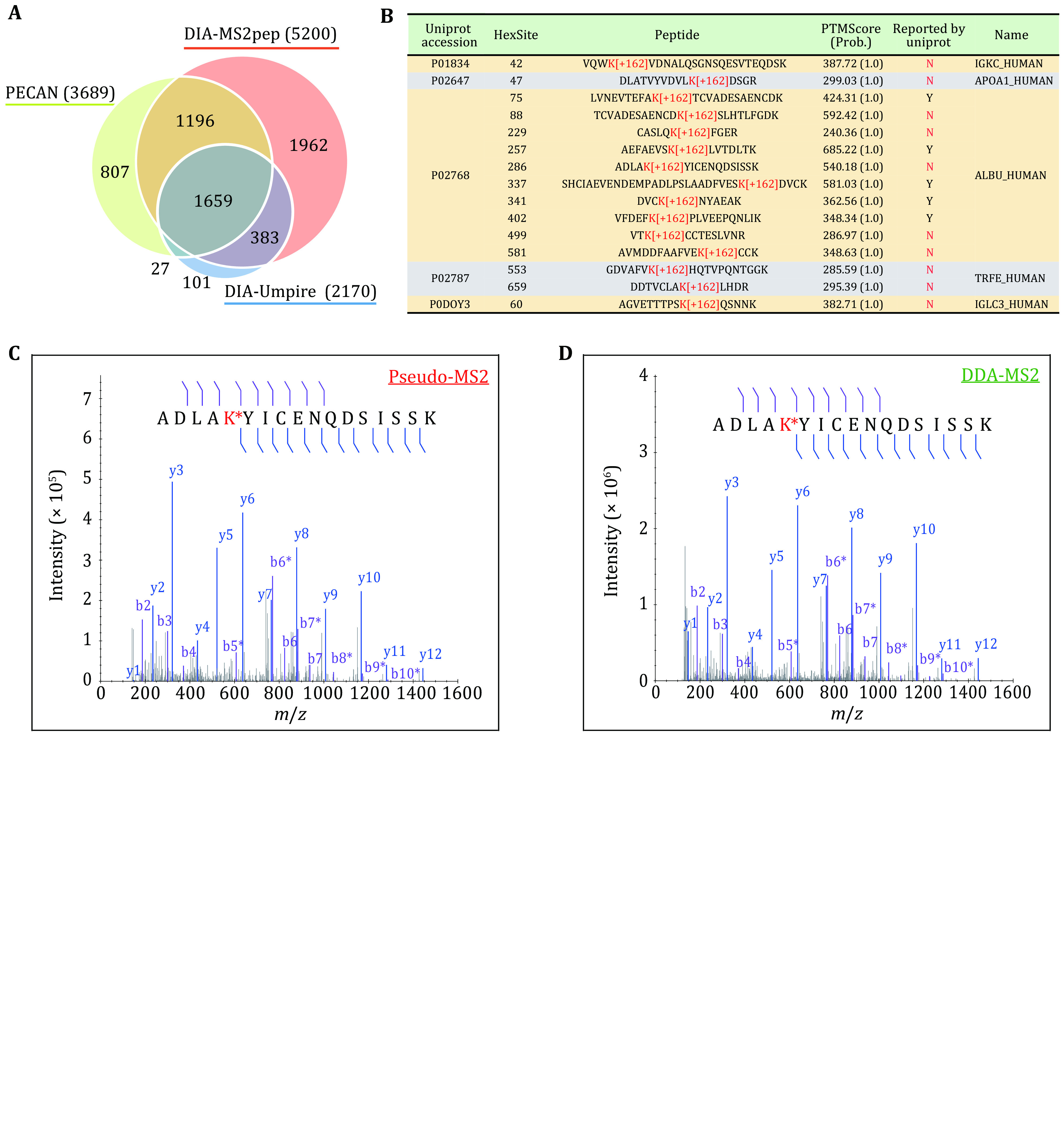
Comprehensive analysis of Plasma_GPF_DIA dataset. **A** The unique peptide number identified by DIA-MS2pep, DIA-Umpire and PECAN from the Plasma_GPF_DIA dataset. **B** Fifteen glycated peptides (Hex[K]) were identified by DIA-MS2pep from the Plasma_GPF_DIA dataset. The sites reported in the UniProt database are marked as “Y”; otherwise, the sites are marked as “N” (red). PTMScores, including site probability as indicated in parentheses, are calculated by DIA-MS2pep to evaluate the site localization confidence. **C**,**D** An example of the DIA-MS2pep pseudo-spectra from Plasma_GPF_DIA dataset (Panel C) vs DDA spectra (Panel D) from the sample of in vitro glycation experiment (supplementary Methods). In the spectra, b- and y-ions are denoted using purple and blue colors, respectively. In addition, the neutral loss peaks of glycation (H_6_O_3_, −54 Da) are also denoted with b* and y* ions

Motivated by the above results, we further explored the discovery potential of DIA-MS2pep using a nondepleted, pooled plasma MS dataset collected by 12 GPF DIA runs (Ting* et al.* 2017) (Plasma_GPF_DIA dataset). In total, DIA-MS2pep identifies 5,200 unique peptides (from 565 protein groups), which is 41% more than PECAN and 140% more than DIA-Umpire, respectively (Fig. 4A), of which 355 peptides are not included in the PeptideAtlas Human Plasma spectral library (2013-08 release).

Interestingly, 15 peptides from five proteins are identified with confidently localized glycation (Hex[K]) (Fig. 4B), which is a non-enzymatic modification of proteins by glucose biologically relevant in the context of obesity and type 2 diabetes (Rhee and Kim 2018). By performing *in vitro* glycation experiment coupled with DDA MS (supplementary Methods), we successfully validate five glycation sites of albumin (ALBU), which are not reported in the UniProt database. As expected, of all five glycated peptides, the DIA-MS2pep pseudo-spectra are highly consistent with the corresponding DDA spectra (Fig. 4C and supplementary Fig. S10), demonstrating the high accuracy of glycation sites identified by DIA-MS2pep. Additionally, DIA-MS2pep detects 332 peptides with putative amino acid variants, of which 64 exist in the UniProt Swiss-Prot human natural variant database (supplementary Table S4). By re-searching the pseudo-spectra generated by DIA-MS2pep from Plasma_GPF_DIA dataset using Mascot with automatic error tolerant search (Mascot-ETS), 290 variants are identified as either consistent (280 peptides) or homologous (10 peptides) sequences (supplementary Table S4).

### Building spectral library from DIA data with DIA-MS2pep

Spectral library for quantitative analysis of DIA data is commonly generated by DDA experiment of pooled samples, while it may be impractical for the case like rare sample quantities in a clinical study. Here, we also explore the potential of the spectral library generated directly from DIA data with DIA-MS2pep using a published DIA dataset of high-complexity proteomes from hybrid species samples 22 (*H. sapiens*, *E. coli*, *C. elegans* and *S. cerevisiae*) with defined quantitative compositions (two samples S1 and S2, 1:1 for *H. sapiens*, 1:1.1 for *C. elegans*, 1:1.2 for *S. cerevisiae*, 1:0.7 for *E. coli*). As a benchmark, we adopt the quantitative data of peptides and proteins reported by using DDA spectral library (DDA_Lib) from the original study 22. From six runs of DIA data, DIA-MS2pep totally identifies 59,365 peptides and 9,134 proteins with *q*-value less than 0.01 at both the peptide and protein levels (supplementary Fig. S11a). Using these peptide identifications, DIA data-specific spectral library (DIA_Lib) is created by Skyline. With EncyclopeDIA (Searle* et al.* 2018), 48,114 peptides and 8,552 proteins are finally quantified (supplementary Fig. S10a). The average changes in the peptides and proteins between S1 and S2 from three replicates are calculated, as illustrated in supplementary Fig. S11b. Comparatively, DIA_Lib can quantify changes in proteins and peptides closer to the theoretical values, while the protein changes of *S. cerevisiae* and *E. coli* are underestimated by DDA_Lib.

### Application of DIA-MS2pep to real biological DIA data

**Figure 5:**
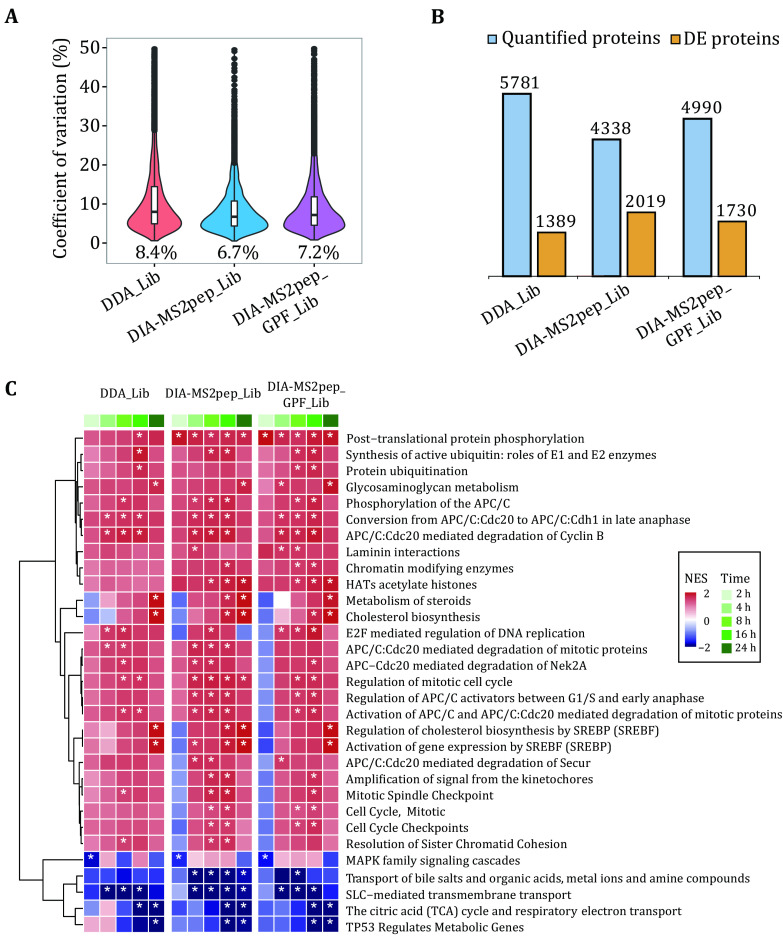
Spectral library built directly from DIA data. Quantitative analysis of the HeLa_Serum_DIA dataset using five different spectral libraries built from either DIA data (DIA-MS2pep_Lib), GPF DIA data plus DIA data (DIA-MS2pep_GPF_Lib) or DDA data (DDA_Lib). **A** The violin combined with a box plot shows the distribution of coefficient of variation (CV) for quantified proteins by different spectral libraries. All box plots indicate the median and IQR, and the whiskers show the 25% and 75% percentiles. The medians of CV are indicated. **B** The number of quantified and differentially expressed (DE) proteins over time with an FDR < 0.01 are reported by edgeR (Lund* et al.* 2012). **C** Heat map of Reactome pathway enrichment analysis using the differentially expressed proteome from the HeLa_Serum_DIA dataset. Pathways with a *p*-value < 0.01 are indicated by asterisks. The Stat. mean values represent the average magnitude and direction of fold changes (the experiment with serum starvation at 0 h was set as the control) at the gene set level of upregulation (red) and downregulation (blue)

Further, we revisit a real biological study that aims to measure HeLa cell proteome changes in response to serum starvation over six-time points (HeLa_Serum_DIA dataset (Searle* et al.* 2018)). In total, DIA-MS2pep identifies 78,265 peptides from six GPF DIA data with narrow windows (52 overlapping 4 *m*/*z*) and 44,233 peptides from DIA data with wide windows (25 × 24 *m*/*z*), and generates two DIA spectral libraries DIA_Lib (wide-window DIA data only) and DIA-MS2pep_GPF_Lib (six GPF DIA plus wide-window DIA data) using Skyline. With the same criteria described in the previous study (Searle* et al.* 2018), we refine the quantitative results as (1) each peptide produced at least three quantitative transition ions without interference, (2) had <20% study-wide CVs, and (3) were measured in every replicate of at least one-time point. Finally, 4,338 and 4,390 proteins are confidently quantified using DIA-MS2pep_Lib, DIA-MS2pep_GPF_Lib, respectively. By comparison, the HeLa-specific DDA library (DDA_Lib) quantifies more proteins (5,781, from the original manuscript of EncyclopeDIA (Searle* et al.* 2018)), but produces less reproducible quantitative data with a higher median coefficient of variation (CV) than the above DIA spectral libraries, as illustrated in (Fig. 5A). The reason for this is likely that, when building the chromatogram library via EncyclopeDIA, peptide information identified by DIA-MS2pep is internally from DIA data itself, while peptide information from DDA data by offline fractionation is different from the real scenario in DIA data, due to the differences of sample matrix and isolation window setting. Using EDGE (Lund* et al.* 2012) to perform differential expression analysis, it is not surprising to observe that the quantitative proteome of all DIA spectral libraries reports more differentially expressed (DE) proteins (*q*-value < 0.01) than that of DDA_Lib (Fig. 5B). Among them, DIA-MS2pep_Lib and DIA-MS2pep_GPF_Lib report more DE proteins, especially those of moderate abundances (supplementary Fig. S12a). Further gene set enrichment analysis (GSEA) (Sergushichev 2020) of the quantitative proteome shows that DIA libraries enable us to reveal more starvation-relevant biological events with significant enrichment than DDA_Lib, such as “Cell Cycle Checkpoints”, “Chromatin modifying enzymes” and “HATs acetylate histones” (Fig. 5C).

In addition, DIA-MS2pep identifies 1,683 peptides with chemical or biological modification or amino acid variants (supplementary Fig. S12b), such as protein N-terminal acetylation, phosphorylation, carbamylation and deamination.

#### Protein N-term acetylation

The most abundant modification identified from the HeLa_Serum_DIA dataset by DIA-MS2pep is protein N-terminal acetylation. In total, 236 unique sequences (based on the first six amino acids in the peptide sequence) with N-terminal acetylation containing a 190 N-terminal methionine acetylation (+42 Da) and a 46 N-terminal methionine cleavage (–89 Da) are identified, of which 196 have been reported in previous literature (Helbig* et al.* 2010). Not surprisingly, we find that most of the acetylated N-terminal amino acids are considered substrates of N-terminal acetyltransferase A (NatA) (supplementary Fig. S13). From the quantitative analysis, 53 N-terminal methionine acetylation and 14 N-terminal methionine cleavage show significant changes over time (*q*-value < 0.01).

#### Phosphorylation

We identify 139 localized phosphopeptides, of which 43 quantitatively responded to serum starvation (*q*-value < 0.01, supplementary Table S5). Using NetworKIN (Horn* et al.* 2014) to predict potential upstream kinases (NetworKIN score > 2.0 and NetPhorest score > 0.1), it is reasonable that eight phosphorylation sites on six proteins are recognized as substrates of CDK1, a key cyclin-dependent kinase participating in the progression of cell mitosis (Enserink and Kolodner 2010). In addition, phosphorylation of T366 on the NDRG1 protein (supplementary Fig. S14a), which is predicted as the substrate of SGK1 (serum and glucocorticoid inducible kinase 1), is a known site of cell cycle dependence (supplementary Fig. S14b). Compared with the upregulated abundance at the protein level in response to serum starvation, the opposite changes of phosphorylation likely represent its functional association with the regulation of protein stability, increasing interest in further follow-up validation.

#### Dimethylation

Three Arg dimethylation sites on three proteins are identified: HNRNPA0 (SNSGPYR[+28.0313]GGYGGGGGYGGSSF), HNRNPA1 (SGSGNFGGGR[+28.0313]GGGFG

GNDNFGR), and RBM3 (SYSR[+28.0313]GGGDQGYGSGR) (supplementary Fig. S15a, S15c and S15e). All three sites are associated with repeated RGG motifs, known as RNA-binding motifs (Kiledjian and Dreyfuss 1992). The quantitative changes over time are either coordinated with protein changes or not (supplementary Fig. S15b, S15d and S15f), likely indicating the functional diversity of regulation by protein methylation.

#### Myristoylation

Interestingly, one myristoylation site (G[+210.2]QSQSGGHGPGGGK) (supplementary Fig. S16a) is identified on the protein PSMC1 (26S proteasome regulatory subunit 4), and the upregulated quantitative changes over time of this site are very close to the protein changes (supplementary Fig. S16b), suggesting that this myristoylation site may act as a constitutive modification for PSMC1 in protein–protein and protein–membrane interactions (Wang* et al.* 2007).

#### Amino acid variants

In total, 292 peptide variants are identified, of which 42 variants are reported in the UniProt Natural Variant database. For example, the peptide KEEENASVI-[-12.0]DSAELQAYPALVVEK of the DNA-dependent protein kinase catalytic subunit (PRKDC) acts as a molecular sensor for DNA damage and has an identified known amino acid variant Ile3434Thr (rs7830743), as observed by a mass shift of –12.0 Da and localized by MS/MS manual inspection (supplementary Fig. S17a). Quantitative time-course changes of PRKDC at the peptide level are highly coordinated with those at the protein level (supplementary Fig. S17b). Likewise, one known amino acid variant (Asp490Glu, rs1049434) of monocarboxylate transporter 1 (SLC16A1) is identified as the peptide AAESPDQKDTD[+14.0]GGPKEEESPV (supplementary Fig. S17c), which also quantitatively changes over time with the same trend as the protein (supplementary Fig. S17d).

## CONCLUSION

We have demonstrated that DIA-MS2pep is a library-free tool for comprehensive peptide identifications and their modified forms from DIA data. DIA-MS2pep introduces three main methodologies specific to improving peptide identification ability: (1) DIA-MS2pep offers a new data-driven algorithm, in which DIA MS/MS spectra can be effectively demultiplexed by learning from fragment data itself (base peak-fragment correlation) without the need of precursor data. More generally, the strategy of spectrum self-demultiplexing is expandable to any type of DIA data including dia-PASEF (Meier* et al.* 2020) and FAIMS-DIA (Bekker-Jensen* et al.* 2020b), and the concept of intra-fragment correlation in principle can be introduced to quantitative analysis of DIA data as well. (2) DIA-MS2pep interprets pseudo-spectra using a large precursor mass tolerance database search by simply assigning center mass of the isolation window as precursor without the need of one specific precursor mass. We demonstrate that this strategy coupled with rigorous data refinement dramatically improves the identification rate of the pseudo-spectra generated from DIA data. Since an uncertain precursor signal for detectable fragments is a common scenario for DIA data, we think our strategy represents an efficient strategy for data interpretation of pseudo-spectra generated from DIA data. (3) DIA-MS2pep allows to identify the peptides with PTMs without the need to pre-define the modifications, and it also confidently localizes the modification position using the high-quality pseudo-spectra. Currently, our strategy allows one modification, and high quality detectable precursor is required; therefore, the next development phase will be dedicated to expanding the suitability of DIA-MS2pep for PTM analysis.

We have evaluated the performance of DIA-MS2pep in the application of building a spectral library directly from DIA data. The number of peptides and proteins quantified by the DIA data-specific library is lower than that quantified by the DDA spectral library, but the accuracy and reproducibility of quantification for the peptides and proteins are higher, which is particularly beneficial for differential expression analysis. More importantly, spectral library building directly from DIA data is straightforward and economic compared with that building with DDA data, and there is no bias of instrument type or fragmentation mode compared with *in-silico* spectral library building by deep learning (Gessulat* et al.* 2019; Yang* et al.* 2020), thus, it is promising for analysing DIA MS-based studies on large cohorts of samples.

DIA-MS2pep is an open-source tool and is well compatible with DIA data from a variety of acquisition modes, instrument types, and downstream DIA quantification tools (for example, EncyclopeDIA and Skyline). The methodological and computational framework introduced in DIA-MS2pep may be feasibly adapted to take advantage of new approaches and technological improvements to DIA data. Taken together, we think DIA-MS2pep, acting as a spectrum-centric method, enables to expand the current library-free toolbox for DIA MS.

## Conflict of interest

Junjie Hou, Jifeng Wang, Fuquan Yang and Tao Xu declare that they have no conflict of interest.
